# Factors Affecting Human Visual Behavior and Preference for Sneakers: An Eye-Tracking Study

**DOI:** 10.3389/fpsyg.2022.914321

**Published:** 2022-06-13

**Authors:** Zihao Chen, Wenfang Song

**Affiliations:** School of Art and Design, Guangdong University of Technology, Guangzhou, China

**Keywords:** visual behavior, preference, eye-tracking, sneaker design, education background, gender effect

## Abstract

Human visual behavior on a product significantly affects their purchasing behavior during online shopping. In this study, two experimental studies were performed to investigate human visual behavior and preference for sneakers using an eye tracking technology. The first study discovered that shoelace and vamp areas of interests (AOIs) attracted more attention than the other AOIs. The second study explored the factors affecting human behavior on sneakers, which employed 30 students from different professional backgrounds (i.e., such as fashion and non-fashion disciplines), and examined 24 sneakers, i.e., combinations of four shoelace styles and six vamp materials. The results showed that both genders irrespective of their professional backgrounds were more concerned about the shoelaces than vamps. The shoelace AOI gained more attention of females than males, while the vamp AOI was more concerned by males than females. The vamp AOI was more concerned by non-professionals than professionals, while the shoelace AOI was paid more attention by professionals than non-professionals. Besides, flat or round shoelaces, canvas, and cow leather or cotton flannel vamp materials were more preferred by the participants than the other types. The findings are of great help for the fashion product designers, the manufacturers, and the sellers to provide the product required by the customers.

## Introduction

In recent times, e-commerce has become the mainstream of shopping channel. In 2020, global retail e-commerce sales reached up to US$3.9 trillion (Statista, 2021a), accounting for 24% of China's total retail sales (Statista, 2021b). Sneakers are one of the most popular products, especially favored by young people (Lindsay-Prince, 2013; Matthews et al., 2021). Young consumers usually make a purchase decision based on the pictures and the text introduction of a product, and the pictures are found to exert a more important role (Kim and Lennon, 2008). Therefore, to increase online sales, it is important for fashion designers, product photographers, and web designers to know how the e-consumers view and interpret pictures of sneakers.

As visual attention on product pictures significantly affects consumers' decision-making action (Milosavljevic and Cerf, 2008), it is important to investigate the factors influencing human visual behavior and preference for a product. It is consistently found that product cues and human factors are the two main influencing factors (Berlyne, 1971; Yang et al., 2002; Karjalainen, 2003; Herpen and Trijp, 2011). The product cues involve two aspects, i.e., experience attributes (e.g., material, texture, thickness, and softness) and search attributes (e.g., shape, color, and size) (Wright and Lynch, 1995; Caswell, 2002; Overmars and Poels, 2015). Lin and Wei (2016) discovered that the bodies of fragrance bottles with sphere shapes and textured surfaces were more preferred by the participants. Fenko et al. (2010) discovered that the wool material was scored higher than the other materials for the pleasantness of scarves. Moreover, studies found that compared to viewing a static product image, using a hand simulating to touch a product image (e.g., a scarf) could significantly increase the perceived diagnosticity of the experience attributes of a product (Overmars and Poels, 2015). Regarding the influence of human factors, scholars found that there exist gender differences in visual attention to a picture. Holbrook (1986) detected that females were more visually oriented and more intrinsically motivated than males. Darley and Smith (1995) discovered that females were comprehensive information processors attracted by subtle cues, while males were selective information processors ignoring subtle cues. In addition, it was also reported that there were gender differences in saccade path between the area of interests (AOIs), and females presented more explorative fixating behavior, longer scan paths, and faster scan speed than males when viewing a picture of the indoor environment (Sargezeh et al., 2019). Besides, the visual behaviors of people from various professional backgrounds are different. Park et al. (2012) revealed that the trained viewers (e.g., designers) exhibited a longer total duration time and more numbers of fixation points than the untrained viewers. Nodine et al. (1993) found that compared with artists, the untrained viewers tended to be more attracted by the representational issues instead of the perceptual organizing function of symmetry.

For investigating consumers' preferences and perceptions of a product, various methods were employed. The semantic differential (SD) method was a widely applied method, which adopted a semantic rating scale measuring the consumers' feelings and attitudes toward the products (Osgood et al., 1957; Guo et al., 2016; Kim and Yun, 2018). However, this subjective assessment suffers from several limitations, such as the relatively small number of subjects employed, difficult description for the subtle and sensitive impression of the products in words (Ho and Lu, 2014), and hard expression of the emotions elicited by product features (Qu and Guo, 2019). As a result, objective methods such as eye-movement tracking, electroencephalography (EEG), and electromyography (EMG) technologies could accurately measure humans' original emotions by detecting their physiological signals when viewing a product. For example, Ho (2014) utilized eye-tracking technology to examine the AOIs of handbags and found that the handle AOI gained more visual attention of females than the other AOIs. Guo et al. (2016) explored the relationship between users' eye movements and their experience level when looking at smartphones, revealing that the users with high-level experience showed shorter first fixation time, longer total fixation time, and larger pupil diameter than those with low-level experience. Furthermore, using both EEG and eye-movement data obtained, Li et al. (2017) applied a fuzzy comprehensive evaluation model to quantify the evaluation factors affecting the shirt appearance of females.

In view of the above, a series of studies were performed on the various product designs using objective methods. However, the research on human visual attention and preference for sneakers gained little attention. To provide solid recommendations for sneaker design, this study aims to explore the factors affecting human visual attention and preference for sneakers online using eye-tracking technology. Two experimental studies were conducted. In the first experiment (Experiment 1), the two most attractive AOIs of sneakers were selected, i.e., the shoelace and the vamp AOIs, and in the second experiment (Experiment 2), the effects of shoelace styles and vamp materials on human visual behavior and preference were examined. It was noted that to simplify the experimental design, the other factors such as color, size, thickness, and softness of the sneakers were not discussed. In addition, the effects of gender and professional background on human visual behavior and preference for sneakers were also investigated.

## Experiment 1: Main Area of Interests

### Methods

#### Participants

A total of 30 University students were recruited in this study, including 12 professionals (6 males and 6 females) and 18 non-professionals (9 males and 9 females). It is noted that the 12 professionals are specialized in fashion design, having received more than 2-year's professional training, and rated high by their course teachers, and the 18 non-professionals were from non-design disciplines, having no understanding of design. Their average (with standard deviation) age, height, and weight were 21.55 ± 1.44 years, 165.85 ± 7.63 cm, and 56.5 ± 8.56 kg, respectively. It was noted that the participants had no vision problems such as short sight, color blindness, and color weakness. In addition, to avoid the possible effect of cultural backgrounds on the experiment, the selection of participants was such that all participants grew up in the Guangdong province of China.

#### Stimuli

To distinguish the AOIs of sneakers, extensive research was conducted to select the sneaker pictures from well-known websites, namely, Amazon, Alibaba, Taobao, and Jingdong. The extensive search yields a great number of potential sneaker pictures, and the pictures were included according to the following criteria based on the objective of this study: (1) The sneakers should be popular online, among the top 50 shoes on the four websites; (2) The sneakers with famous logos (e.g., Nike brand) or the well-known famous design features (e.g., Adidas' three horizontal stripes) were excluded; (3) The sneakers should be displayed at approximately 45° from the horizontal line to be more stereoscopic and realistic; (4) The sneakers should be diverse in color, shape, material, and design details; (5) The sneakers were unisex.

After the selection, a total of 200 sneakers were included. Further selection was performed by two experts who have more than 8-years of experience in the fashion industry and two college teachers who have more than 10-years of experience in fashion design. Finally, 18 sneaker pictures were selected for further study.

#### Apparatus

The eye-movement data of participants were captured by Eye Link 1000 plus (SR research, Canada) and the sampling rate was 1,000 Hz. The 18 sneaker images were edited and displayed in E-Prime Professional 3.0 software. During the tests, the images were randomly presented in the center of a 21-inch Liquid Crystal Display (LCD) monitor with a resolution of 1,920 × 1,080 pixels. Noteworthy is that the sneaker had a width of 20.5 cm and was located in the middle of the image, taking up two-thirds of the image area. The background of the image was gray (Red Blue Green (RBG): 101, 101, 101). Data viewer software (SR research, Canada) was adopted to capture the interest areas and analyze eye-movement data. It was noted that the experiment was conducted in a closed quiet room with good lighting conditions. The air temperature and relative humidity (RH) in the room were controlled at 26°C and 50% RH, respectively.

#### Procedure

Upon arrival at the testing room, the participants sat on a chair and put on their chins on the handle fixed on a desk. Then, the height of the handles was adjusted to ensure that the eyes of the participants were facing the center of the screen in a comfortable posture. Before each formal test, calibration was required to ensure the accuracy of the eye-movements data captured, in which the participant followed “+” signs which emerge sequentially in the specified five positions of the screen.

**Figure 1:**
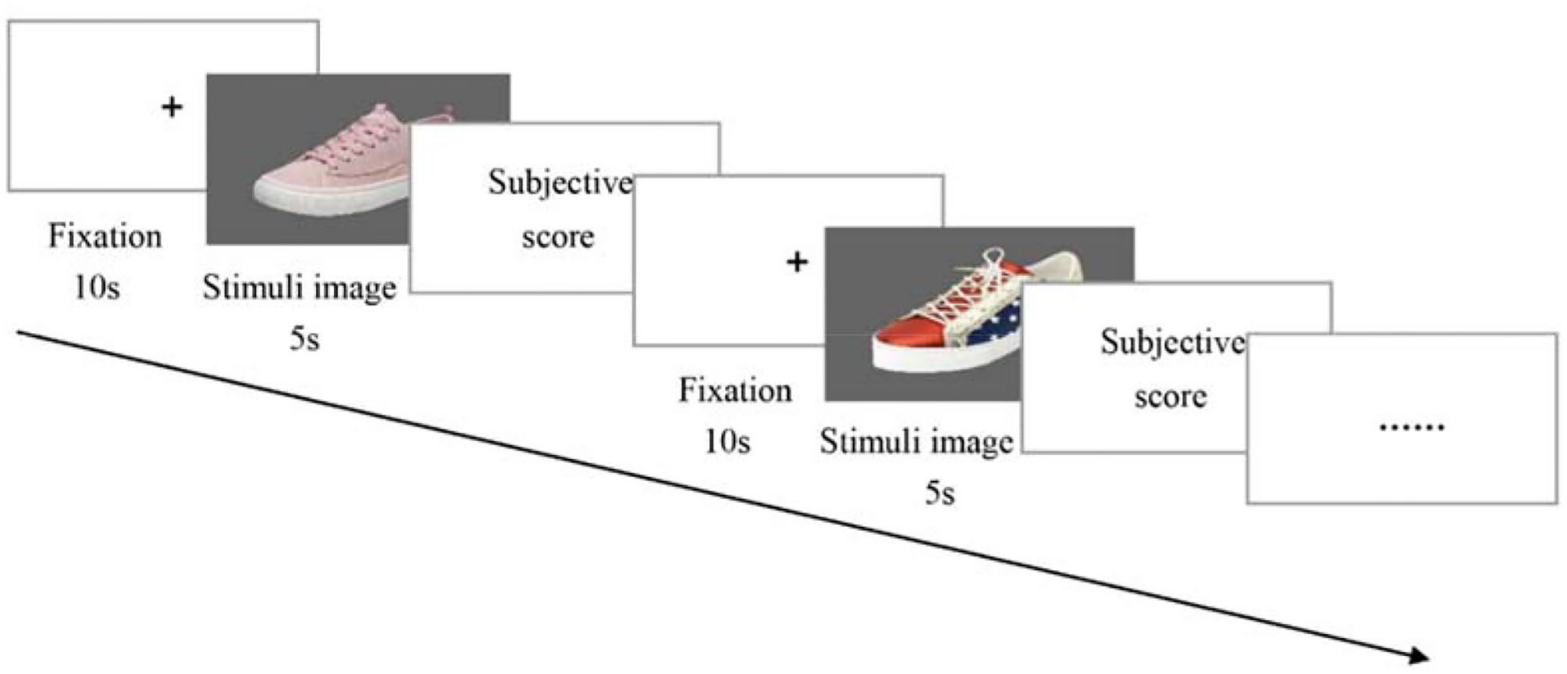
The testing procedure of the sneakers.

**Figure 2:**
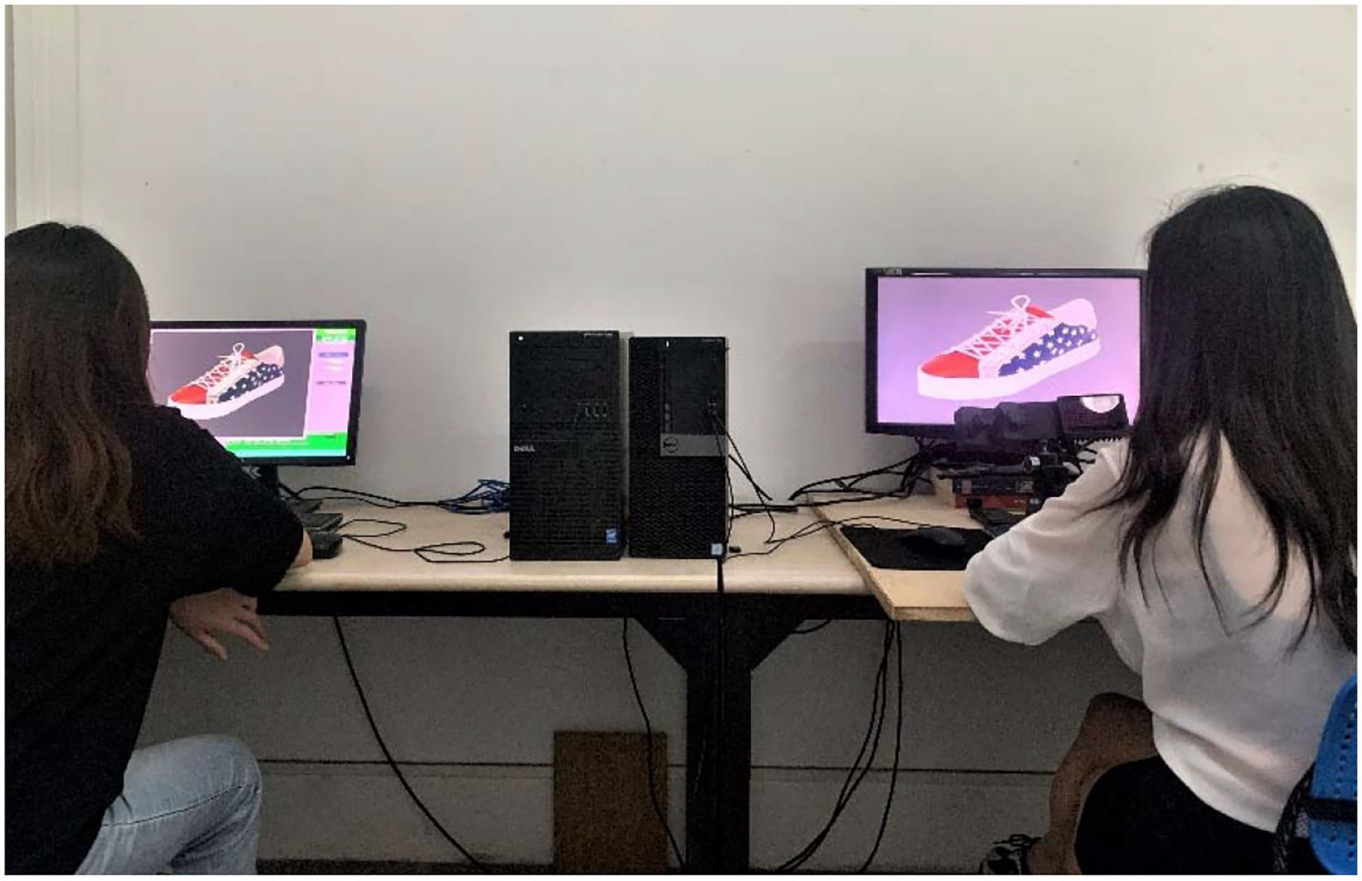
An image of the experiment.

During the tests, the participants consistently looked at the “+” sign at the center of the screen for 10 s until the stimulus appeared. Then, each sneaker image was presented for 5 s. After that, the participants rated their preference for the sneakers using a 5-point scale, ranging from 1 (“dislike very much”) to 5 (“like very much”) (Chai et al., 2015). The above procedure was repeated until all sneaker images were examined (Figure 1). It was noted that all images were randomly displayed on the screen to minimize the influence of physiological factors such as visual fatigue. In addition, to help the participants fully understand the experimental process, a pre-test using two images of sneakers like the selected ones was conducted following the above procedure before each formal experiment, which is not included in the final data analysis. Figure 2 displays the situation of the experiment.

#### Determination of the Areas of Interests

Areas of interests refer to the divided regions before the experiment according to the feature of the research object, and the eye-movement data were captured in these regions, which were used to find the main areas of interest of the object (Ho, 2014).

**Figure 3:**
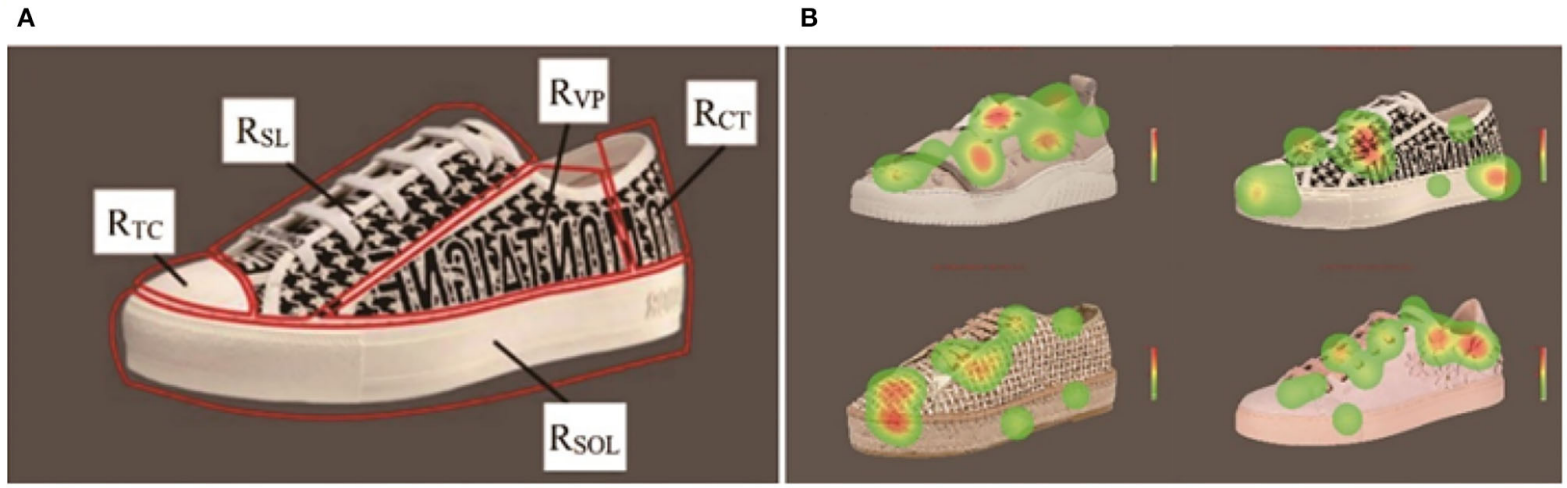
Samples of stimuli sneaker images. **(A)** Display of the AOIs of a sneaker, i.e., toe cap (R_TC_), shoelace (R_SL_), vamp (R_VP_), counter (R_CT_), and sole (R_SOL_). **(B)** Display of the heat maps of four sneakers from a participant for example.

According to the design features of the sneakers, five AOIs were divided, i.e., toe cap (R_TC_), shoelace (R_SL_), vamp (R_VP_), counter (R_CT_), and sole (R_SOL_), shown in Figure 3A. The eye-movement signals collected from these five AOIs could be used to analyze the visual attention of the participants. The total fixation duration and the average number of fixation points were used to detect the AOIs of sneakers that gained more attention of the participants. Figure 3B shows the heat maps of four sneakers from a participant for example.

#### Statistical Analysis

A one-way ANOVA was carried out to analyze the differences in eye-movement signals of the participants between any two of the five AOIs, which was corrected with Bonferroni multi-comparison. All data were analyzed using SPSSv.22 (IBM Inc., USA). The differences were presented as *p*-values and the statistically significant differences were set as *p* < 0.05 and *p* < 0.01, marked as “^*^” and “^**^,” respectively.

## Results and Discussion

Table 1 shows the one-way ANOVA analysis results of the eye-movement signals. It was clearly seen that there were significant differences in both total fixation duration [*F*_(4,2655)_ = 776.42, *p* < 0.001] and the average number of fixation points [*F*_(4,2655)_ = 845.78, *p* < 0.001] among the five AOIs.

**Table 1:** The descriptive statistics and results of repeated measures ANOVA for an eye-tracking index in Experiment 1.

**Eye-Tracking Index**	**R_**TC**_**	**R_**SL**_**	**R_**VP**_**	**R_**CT**_**	**R_**SOL**_**	***F***	***p***
Total fixation duration (ms)	184.08 (273.88)	1,739.01 (904.32)	1,569.96 (845.60)	281.47 (414.94)	324.90 (479.37)	776.42	0.000**
Average number of fixation points	0.73 (1.10)	6.43 (3.18)	6.01 (3.01)	1.03 (1.43)	1.28 (1.84)	845.72	0.000**

*The data were the average of all measures of all subjects and were expressed as mean (standard deviation)*.

Specifically, significantly longer total fixation duration was observed in R_SL_ (1,739.01 ± 904.32) than R_VP_ (1,569.96 ± 845.6) (*p* < 0.001), R_SOL_ (324.90 ± 479.37) (*p* < 0.001), R_CT_ (281.27 ± 414.94) (*p* < 0.001), and R_TC_ (184.08 ± 273.88) (*p* < 0.001), and R_VP_ showed longer fixation duration than R_SOL_, R_CT_, and R_TC_ (*p* < 0.001). In addition, significantly larger numbers of fixation points were detected in R_SL_ (6.43 ± 3.18) than R_VP_ (6.01 ± 3.18) (*p* = 0.027), R_SOL_ (1.28 ± 1.84) (*p* < 0.001), R_CT_ (1.01 ± 1.43) (*p* < 0.001), and R_TC_ (0.73 ± 1.10) (*p* < 0.001), and R_VP_ showed larger numbers of fixation points than R_SOL_, R_CT_, and R_TC_ (*p* < 0.001). The results indicated that the shoelace AOI (R_SL_) and shoe vamp AOI (R_VP_) received more attention than the other AOIs.

Previous studies found that human beings tended to be attracted by the feature and brand of a product (Pieters and Warlop, 1999; Corbetta and Shulman, 2002; Ho, 2014; Laan et al., 2015). Normally, the shoelace and vamp of the sneakers contained more design features than the other regions. Specifically, the design features in the shoelace include color, a contrast of texture, brands, etc.; those in the vamp involve material, brands, text, patterns, etc.; and those in the other regions often include materials and patterns. It is thus speculated that the shoelace and vamp attracted more attention than the other AOIs due to the more design features in the two regions. The finding may be consistent with that of Ho (2014) who found that people had more interest in the handle region of the handbag which owns more design features than the other parts.

Another possible reason is that the areas of shoelaces and vamp were larger than the other AOIs due to the display of the sneaker images (Figure 2), resulting in longer fixation duration and a greater number of fixation points. This does not negate the experimental design, as the display of the sneakers was the main way of the presentation online.

## Experiment 2: Main Factors Affecting the Visual Esthetic Preference

### Methods

#### Participants

In this study, 30 Chinese University students (15 males and 15 females) were employed. Assuming an effect size *f* = 0.4, α = 0.05, and power (1-β) error probability of 0.95, 12 participants would provide enough power to detect a significant difference (G^*^Power, version 3.1.9.2), indicating that the number of participants employed here is enough. It is noted that 14 participants (7 males and 7 females) are professionals, and 16 participants (8 males and 8 females) are non-professionals, and the definition of the “professional” is the same as that in Experiment 1. The age of the participants ranges from 18 to 22 years (20.74 ± 1.19 years). The participants had no vision problems and no history of taking drugs or alcohol.

#### Stimuli

**Figure 4:**
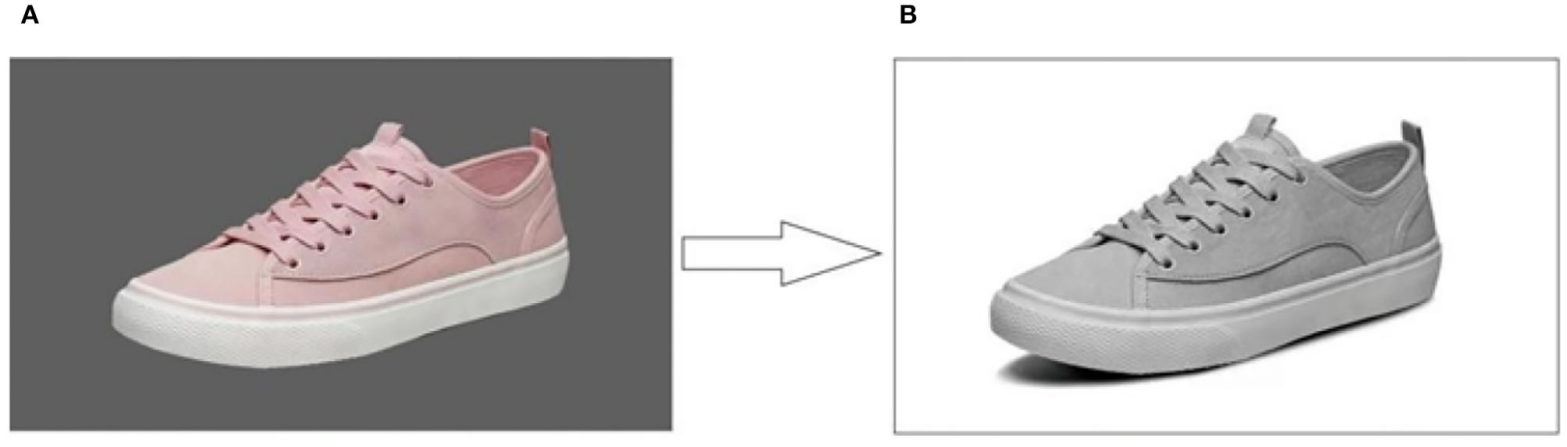
The chosen sample for further design. **(A)** The original picture. **(B)** The picture after the removal of color.

**Figure 5:**
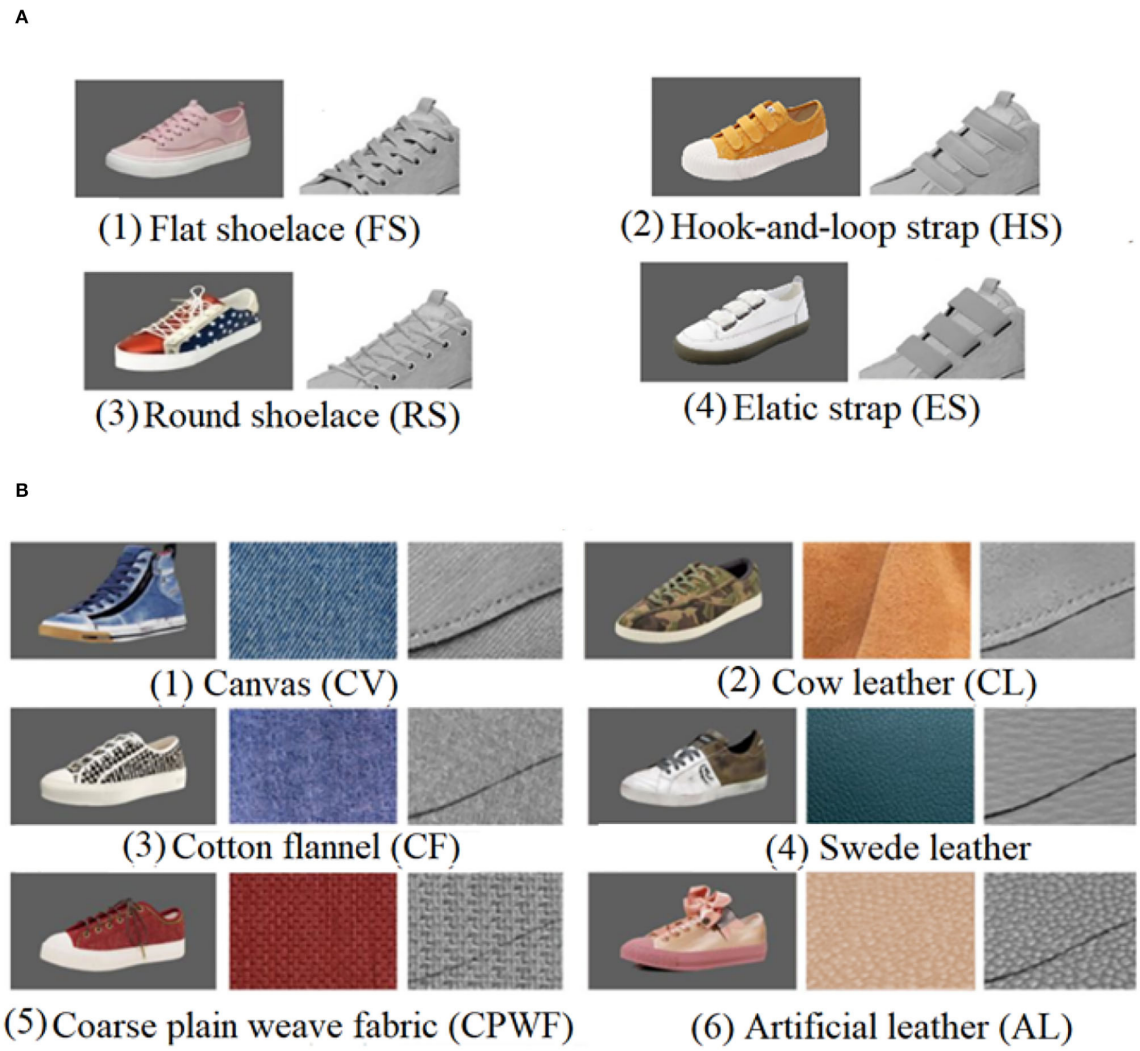
Typical shoelaces and vamp materials obtained from the websites. **(A)** The four types of shoelaces. **(B)** The six types of vamp materials.

As observed in Experiment 1, the shoelace (R_SL_) and vamp (R_VP_) of sneakers were the two main AOIs, and thus examined for their design features in affecting the human's visual behavior and preference. It was noted that the sneaker most favored by participants was selected as the sample to be designed (3.52 ± 0.78) (Figure 4). Four typical types of shoelaces, i.e., flat (FS), round (RS), hook-and-loop strap (HS), and elastic strap shoelaces (ES) (Figure 5A), and six types of vamp materials, i.e., canvas (CV), cotton flannel (CF), coarse plain weave fabric (CPWF), cow leather (CL), swede leather (SL), and artificial leather (AL) (Figure 5B), were combined to produce 24 sneaker designs. Noteworthy is that the rest three AOIs of the sneakers remained the same for the 24 images. It was also noted that the shoelaces and vamp materials were extracted from the images on the websites and processed by Photoshop CS6 (Adobe Systems Inc., United States). After the removal of colors, the sneakers turned gray, and the backgrounds were white. The size of the images, the position of the stimuli on the screen, and the size of the stimuli were kept the same as those in Experiment 1.

#### Apparatus and Testing Procedure

The eye-tracking apparatus, the image display, and the data acquisition software were the same as those used in Experiment 1. In addition, the testing procedure here followed that described in Experiment 1. Noteworthy is that the sneakers for stimuli were the 24 sneaker images, a combination of the four types of shoelaces and six types of vamp materials (Figure 5).

#### Eye-Tracking Measures

The aim here was to examine the effects of the shoelace and vamp design features, the genders, and professional backgrounds on human visual behavior and preference for sneakers. Hence, the total fixation duration, the average number of fixation points, and average pupil size were used for the assessment. These eye-movement indexes were widely used to assess the attractiveness of particular AOIs (Ho, 2014) and the level of concern or preference for particular AOIs (Qu and Guo, 2019).

#### Statistical Analysis

One-way ANOVA corrected with Bonferroni multi-comparison was used to analyze the differences in eye-movement data among genders, professional backgrounds, styles of shoelaces, and materials of vamps. The *p*-values, “^*^” and “^**^” have the same meanings as those in Experiment 1.

### Results and Discussion

#### The Effects of Genders

**Figure 6:**
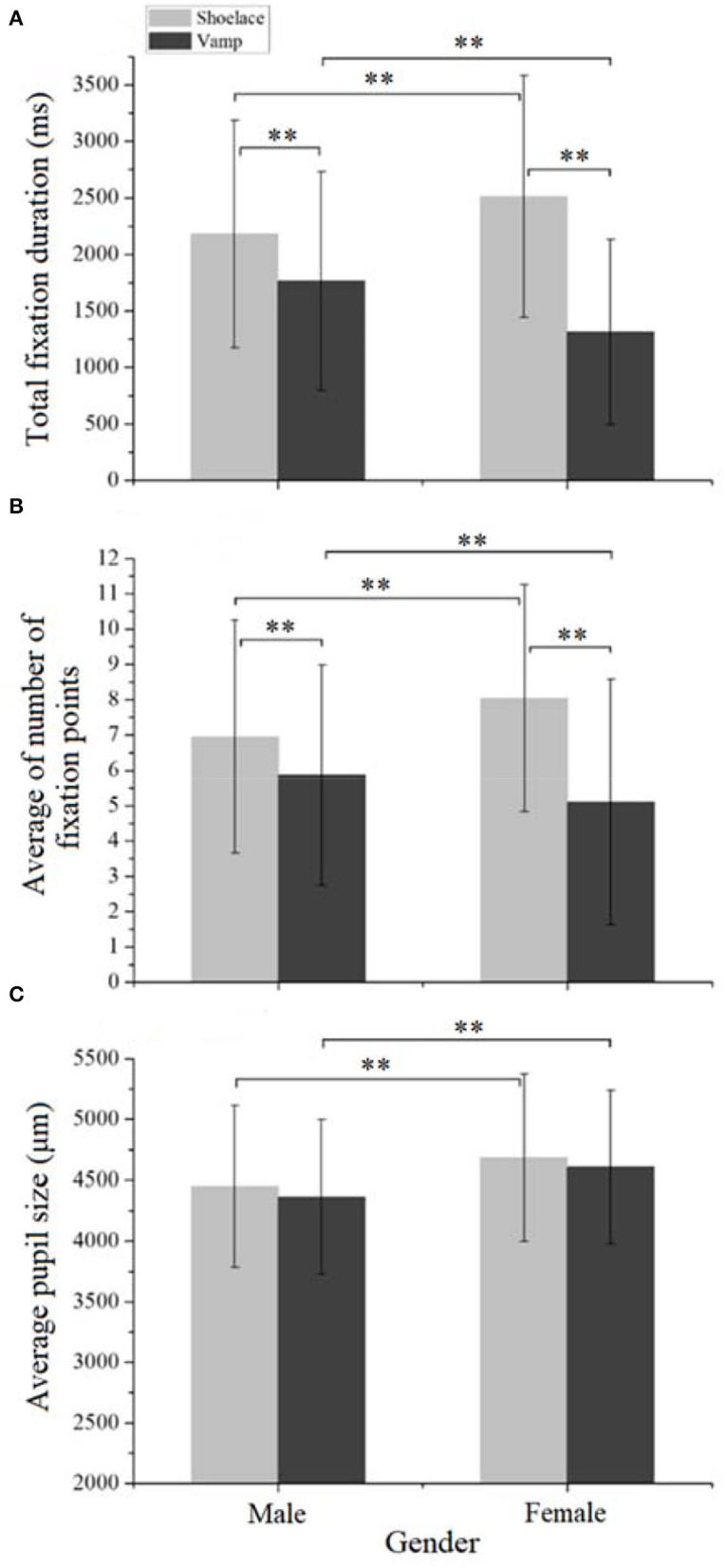
Gender difference in eye-movement data. **(A)** The total fixation duration. **(B)** The average number of fixation points. **(C)** The average pupil size.

Figure 6A shows the effect of genders on the total fixation duration of the participants. The significant differences were found in the total fixation duration between genders in the shoelace AOI [*F*_(1,683)_ = 17.60, *p* < 0.001] and vamp AOI [*F*_(1,683)_ = 42.80, *p* < 0.001]. Specifically, females tend to spend more time in viewing the shoelace AOI than males, while they showed shorter fixation duration in the vamp AOI than the males. The possible explanation is that females were more interested in the appearance of sneakers than males, and less interested in the function of sneakers than males (Brownmiller, 1984). This may be consistent with the findings of the previous studies that reported females are more interested in the design details of a product (Qu and Guo, 2019), and males were more concerned about the functional items rather than the appearance items of a product (Dittmar et al., 1995). For the sneakers, the shoelace AOI may represent an area that requires detailed design, and the vamp AOI may be the main functional area.

It was also discovered that for both males and females, a significant difference in total fixation duration was detected between the two AOIs (*p* < 0.001). They spent more time looking at the shoelace AOI than the vamp AOI (*p* < 0.001). In this study, the shoelace AOI was dominated by the search attributes (e.g., shoelace styles), which were primarily processed by vision. Differently, the vamp AOI was featured with experiment attributes (e.g., material and texture), which were processed by both vision and touch. When shopping online, consumers could not touch the vamp materials, and the gain of experiment attribute information mainly relies on transforming the tactile to vision sense. Some researchers discovered that vision sense can be used in conjunction with, or instead of, the other sensory information exploration to form impressions of objects (Hollier et al., 1999; Yazdanparast and Spears, 2012). However, it is found that transforming tactile to vision sense was not effective to recognize product cues using a still picture. Some researchers found that simulating stroking gestures on a product picture improved the consumers' impression of the experience attributes of a product (Overmars and Poels, 2015). As a result, on the condition of using only a still product picture, the participants tend to spend less time exploring experiment attribute cues than search attribute cues. This may explain the finding here that participants spend more time viewing the shoelace AOI than vamp AOI.

Figure 6B shows the effect of genders on the average number of fixation points of the participants. There was a significant difference in the fixation points between the two genders for both shoelace [*F*_(1,683)_ = 18.51, *p* < 0.001] and vamp AOIs [*F*_(1,683)_ = 9.05, *p* < 0.01]. A significantly greater number of fixation points were found on females than males in the shoelace AOI, while fewer fixation points were detected on females than males in the vamp AOI. This indicates that females searched more frequently than males in shoelaces. The possible explanation is that females tend to respond to subtle cues, while males tend to use heuristics processing and miss subtle cues (Darley and Smith, 1995). This may be consistent with the finding of Sargezeh et al. (2019) who found females exhibited faster speed in examining a product detail than males. Differently, females searched less frequently than the males in the vamp AOI, possibly because they displayed shorter fixation duration in this region. Besides, the significant differences were found in the average number of fixation points between the two AOIs for both males [*F*_(1,716)_ = 20.51, *p* < 0.001] and females [*F*_(1,650)_ = 125.29, *p* < 0.001]. Both males and females viewed more frequently at the shoelace AOI than the vamp AOI. This may be consistent with the previous finding that human beings are more concerned about the appearance of a product (e.g., shape and size), which dominated the shoelace AOI.

Figure 6C displays the effect of genders on the pupil sizes of participants. There were the significant differences in the pupil sizes between the two genders in the shoelace AOI [*F*_(1,683)_ = 20.14, *p* < 0.001] and vamp AOI [*F*_(1,683)_ = 25.31, *p* < 0.001]. Females showed remarkably larger pupil sizes than males in both shoelace and vamp AOIs. No difference was discovered between the two AOIs for both males and females (*p* > 0.05). According to previous studies, human pupillary response to a stimulus reflects their emotional arousal to it (Ho and Lu, 2014). They have large pupil sizes when viewing positive and negative stimuli than viewing neutral stimuli (Bradley et al., 2008), and females were more apt to show larger pupil diameters when viewing pleasant and neutral product images than males (Qu and Guo, 2019). These may explain the finding here that females showed larger pupil sizes than males when observing both shoelaces and vamp AOIs. It indicates that females were more remarkably evoked by the sneakers than males.

However, for the subjective evaluation, a similar score was achieved for females (2.99 ± 0.84) and males (2.92 ± 0.85) for the sneakers, and no significant difference was found between the two genders [*F*_(1,683)_ = 0.94, *p* = 0.33]. The subjective evaluation manifested that the differences in visual attention between the genders did not stir up differences in their preference for the sneakers. The possible explanation is that the sneakers are the common unisex types, and thus no difference in the preference between the genders was observed.

#### The Effect of Professional Backgrounds

**Figure 7:**
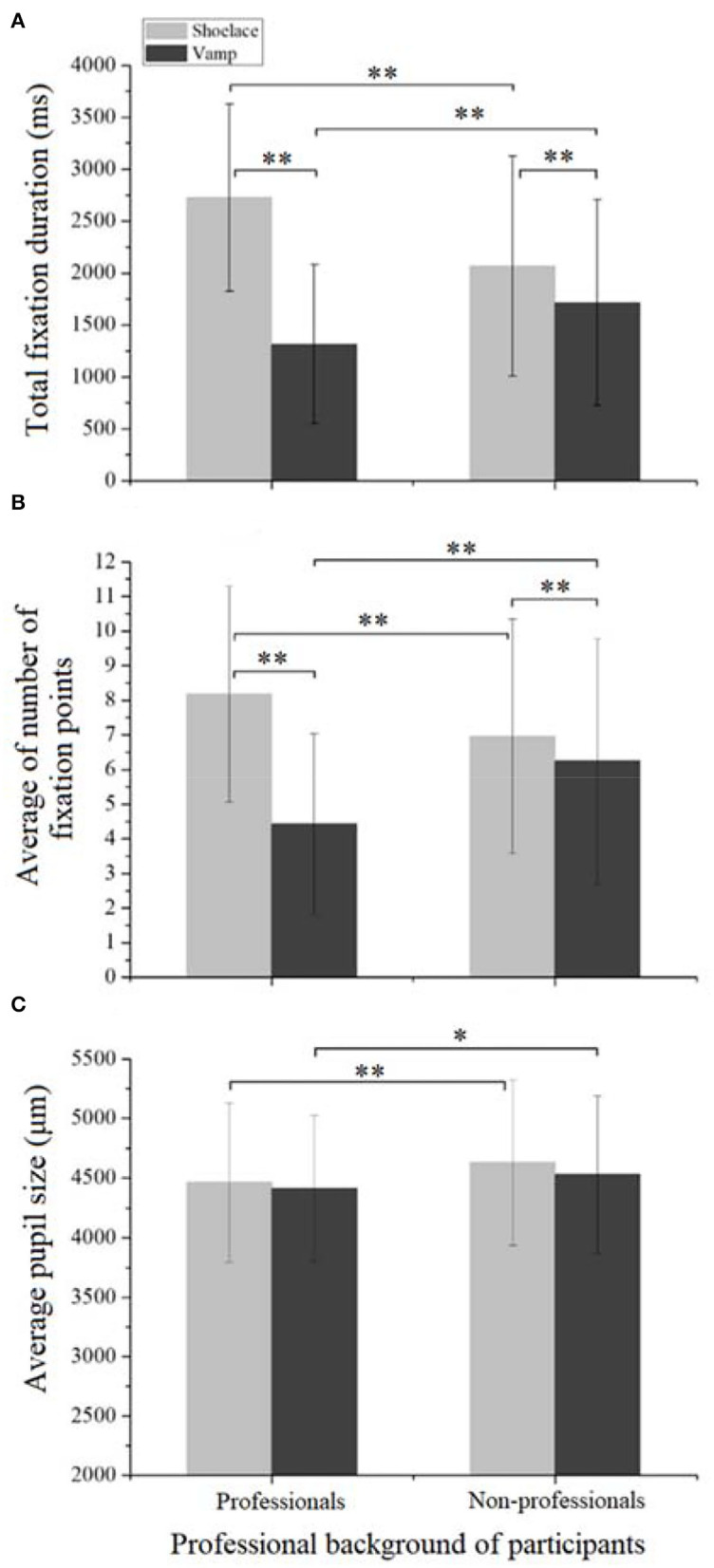
The effect of professional backgrounds on the eye movements of participants. **(A)** The effect on the total fixation duration. **(B)** The effect on the average number of fixation points. **(C)** The effect on the average pupil size.

Figure 7 exhibits the effect of professional backgrounds on the eye movements of participants. There were significant differences in the total fixation duration between the professionals and non-professionals in both shoelace [*F*_(1,683)_ = 71.81, *p* < 0.001] and vamp AOIs [*F*_(1,683)_ = 32.37, *p* < 0.001]. It is found that the professionals spend longer time in viewing the shoelace AOI than non-professionals (Figure 7A). The possible reason is that the professionals were more concerned with design features than the non-professionals because of their professional knowledge backgrounds. This may be consistent with the study of Park et al. (2012) who found that the viewers that received esthetic training were more sensitive to the changes in design details. Differently, the non-professionals displayed a shorter time in looking at the vamp than professionals (Figure 7A). The possible explanation is that compared to the professionals, the non-professionals tended to be more concerned about the functionality of a product. For both professionals and non-professionals, significant differences were found in the total fixation duration between the two AOIs (*p* < 0.001), i.e., they spend more time fixating on the shoelace AOI than the vamp AOI. This may be consistent with the previous studies that human beings were more interested in the appearance of a product.

It is also observed from Figure 7B that the professional background exerted a significant role in affecting the average number of fixation points in the shoelace AOI [*F*_(1,683)_ = 22.77, *p* < 0.001] and the vamp AOI [*F*_(1,683)_ = 53.51, *p* < 0.001). A significantly greater number of fixation points were found for the professionals in the shoelace AOI, while a smaller number of fixation points were discovered on the non-professionals than the professionals in the vamp AOI. The possible explanation is that the professional background of the professionals may make them more attentive to the design details. On the contrary, the non-professionals are more concerned about the functionality of the products. Another possible reason is that compared to the non-professionals, the professionals displayed significantly longer fixation duration in viewing the shoelace AOI and remarkably shorter fixation duration in looking at the vamp AOI. Besides, both professionals and non-professionals showed a greater number of fixation points in the shoelace AOI than the vamp AOI (*p* < 0.001).

Figure 7C displays that the professional backgrounds have significant impact on the average pupil size in the shoelace [*F*_(1,683)_ = 10.05, *p* < 0.01] and vamp AOIs [*F*_(1,683)_ = 5.66, *p* = 0.018]. The professionals were found to have smaller average pupil sizes than the non-professionals when viewing both shoelace and vamp AOIs. This is possibly because the professionals have higher esthetic needs than the non-professionals, and thus their emotion was less affected by the sneakers. Besides the above findings, insignificant differences were found between the two AOIs for both professionals and non-professionals (*p* > 0.05).

In subjective assessment, a similar score was obtained between the professionals (2.94 ± 0.91) and non-professionals (2.97 ± 0.74), and no significant difference was discovered between the two professional backgrounds (*p* > 0.05). Although the differences in visual attention were discovered, no difference in the preference for sneakers was discovered between the two professional backgrounds. The possible explanation is that the sneakers selected are the common design for both professionals and non-professionals, insufficient to cause the difference in their preference for the sneakers.

#### The Effect of Shoelace Styles

**Figure 8:**
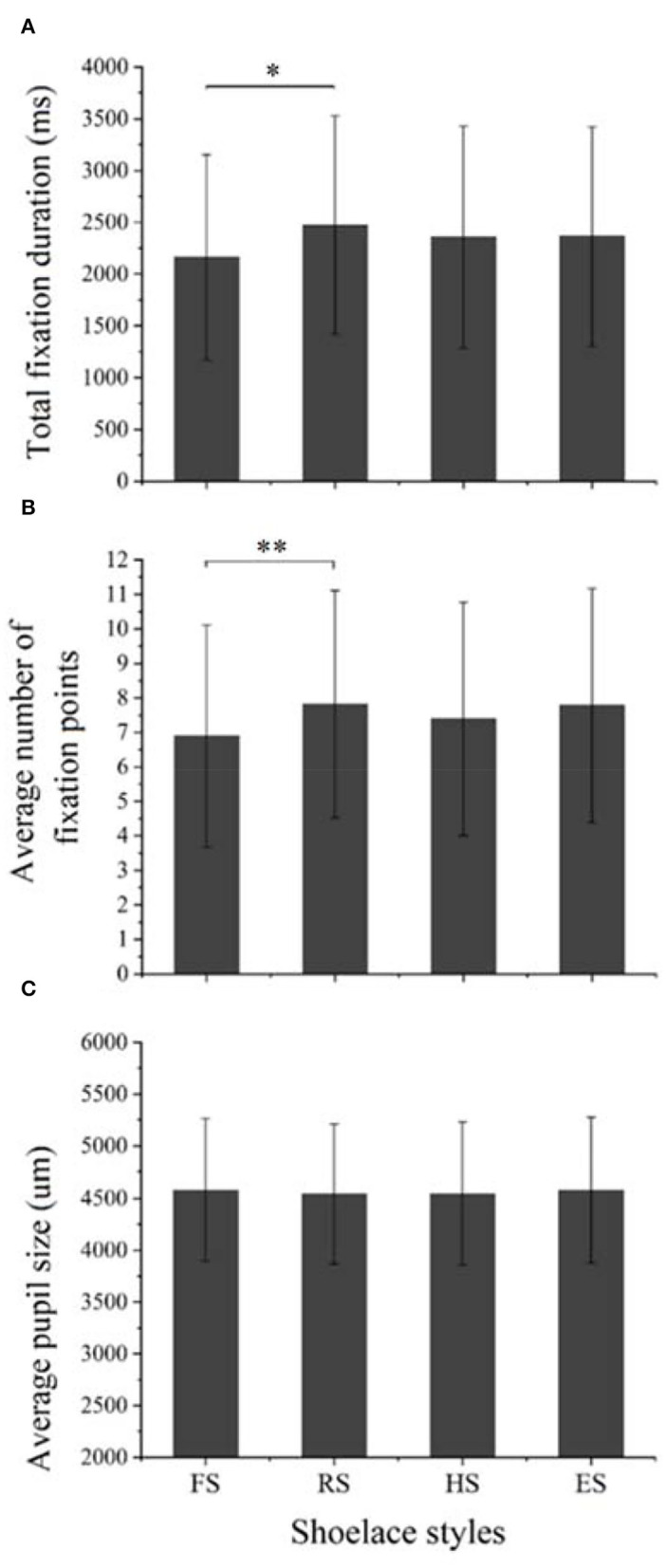
The effect of shoelace styles on eye movements. **(A)** The effect on the total fixation duration. **(B)** The effect on the average number of fixation points. **(C)** The effect on the average pupil size.

Figure 8 displays the effect of shoelace styles on the eye movements of the participants. The significant difference in the total fixation duration was found among the four shoelace styles [*F*_(3,681)_ = 2.61, *p* = 0.05]. The round shoelaces (RS) received a longer fixation duration than flat shoelaces (FS) (*p* < 0.05), and there was no difference among FS, hook-and-loop strap (HS), and an elastic strap (ES) or among RS, HS, and ES (*p* > 0.05) (Figure 8A). Besides, a remarkable difference in the average number of fixating points was detected among shoelace styles [*F*_(3,681)_ = 2.85, *p* = 0.036]. The *post hoc* analysis revealed that RS displayed a greater number of fixation points than FS (*p* < 0.01), and there was no difference among FS, HS, and ES or among RS, HS, and ES (*p* > 0.05) (Figure 8B). Differently, there is no significant difference in the average pupil size among the shoelace styles [*F*_(3,681)_ = 0.16, *p* = 0.92] (Figure 8C). The round shoelace received a longer total fixation duration and a greater number of fixation points than flat shoelaces. The possible explanation is that the round shoelace is fine, and the participants have to spend more time and pay more attention to identify the details.

**Figure 9:**
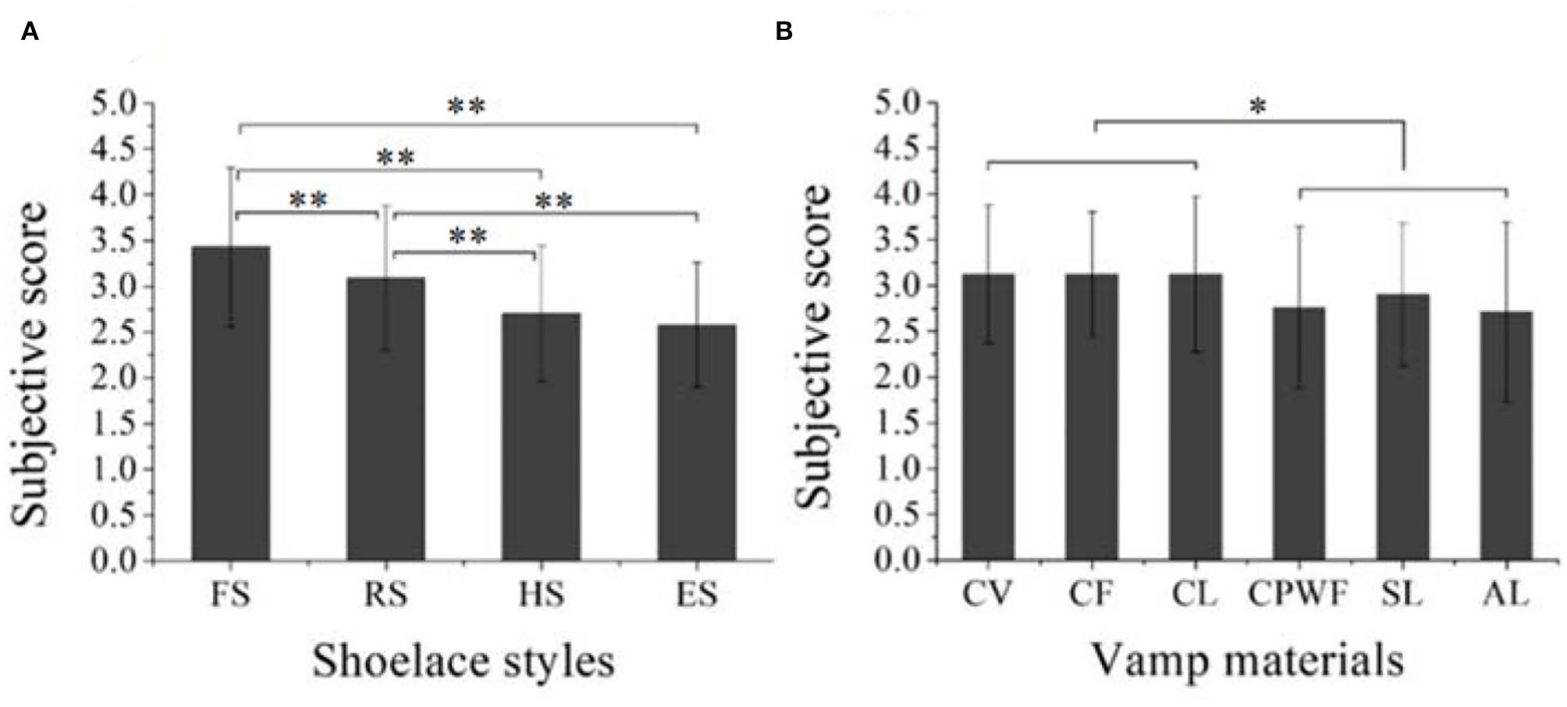
The effect of design features on the preferences of the participants. **(A)** The effect of shoelaces styles. **(B)** The effect of vamp materials.

For the subjective evaluation, a significant difference was detected among the four shoelace styles [*F*_(3,681)_ = 42.96, *p* < 0.001]. Specifically, sneakers with FS were scored higher than those with RS, HS, and ES (*p* < 0.001). Round shoelace was rated higher than HS and ES (*p* < 0.001); HS and ES showed no difference (*p* = 0.13) (Figure 9A). This indicated that the flat and round shoelaces were more preferred than the other shoelace types, and the flat shoelace was the most preferred type. The possible reason is that the two shoelace types are typical in traditional sneakers, which fit the consumers' stereotype for sneakers and thus make it easier for the participants to accept.

#### The Effect of Vamp Materials

**Figure 10:**
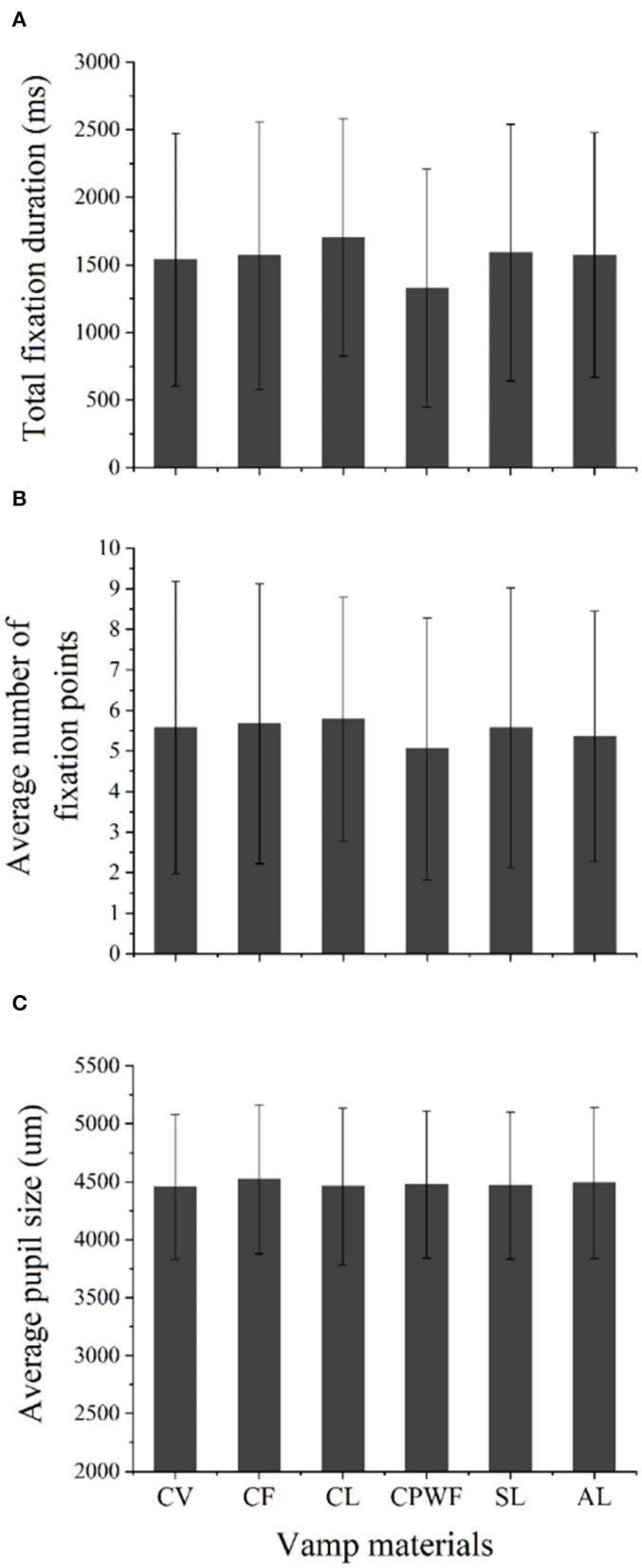
The effect of vamp materials on eye movements. **(A)** The effect on the total fixation duration. **(B)** The effect on the average number of fixation points. **(C)** The effect on the average pupil size.

No significant difference was found in the total fixation duration (*p* = 0.08) (Figure 10A), the average number of fixation points (*p* = 0.62) (Figure 10B), or the average pupil size (*p* = 0.98) (Figure 10C) among the vamp materials. The possible explanation is that the vamp materials selected seem similar, which cannot cause the differences in visual attention of the participants. Another possible reason is that the identification of vamp materials requires more touch than vision, which is hard to distinguish solely based on vision.

For the subjective assessment, the significant differences were detected among the six vamp materials [*F*_(5,679)_ = 6.01, *p* < 0.001]. The sneakers with canvas (CV), cow leather (CL), and cotton flannel materials (CF) were more preferred by the participants than those with coarse plain weave fabric (CPWF), swede leather (SL), and artificial leather materials (AL) (*p* < 0.05). No significant difference was found among CV, CL, and CF or among CPWF, SL, and AL (*p* > 0.05) (Figure 9B). It seems that the vamp materials with smooth surfaces were more preferred by the participants.

### Practical Implications, Limitations, and the Future Studies

In this study, it is found the shoelace and vamp AOIs gained more interest than the other sneaker regions, which are recommended as the key design regions for designers. Both genders, irrespective of their professional background, were more concerned about the shoelaces than vamps, indicating that the design of shoelaces is critical. Besides, females paid more attention to shoelaces than males, while males are more concerned about vamps than females. This indicates that the detailed design or exhibition of sneaker shoelaces is more needed for the female population, and differently, a balance of shoelace and vamp design or exhibition is required for males. Apart from that, the professionals were more concerned about shoelaces than the non-professionals, while the non-professionals paid more attention to vamp materials than the professionals. This indicates that the designers or sellers also need to focus on the design or exhibition of vamps while giving emphasis on shoelaces. As for the design features, flat or round shoelaces, and canvas, cow leather or cotton flannel vamp materials were more preferred than the other types of shoelaces and vamp materials. The study could provide solid guidelines for fashion designers, manufacturers, and sellers to satisfy the demand of the customers.

Several limitations of this study must be acknowledged. This study did not examine the other important factors affecting human visual behavior such as color, logo, and the other design details due to the complexity of the experimental design, and future studies should include these factors. In addition, only a small number of young University students were employed, and future studies should employ a larger sample of subjects and also include different age ranges. Beyond that, the subjects employed are without eye disease, and future tests could be performed with subjects having various types of eye diseases. Finally, this study only discussed the factors influencing human visual attention and preference for sneakers, and future studies will be performed from the perspective of marketing behaviors (such as decision making). The previous studies established the relationship between advertising design, visual attention, and sales using a mediation analysis based on a theoretical model (Wedel and Pieters, 2008; Zhang et al., 2009), which is beneficial for improving the effectiveness and efficiency of advertising decisions. From these studies, we will explore the relationship among the influencing factors (image size, design feature, gender, professional background, price, etc.), human visual attention, and their marketing behaviors to improve the design or advertising of sneakers.

## Conclusion

For the first time, the factors affecting human visual attention and preference for sneakers were investigated using eye-tracking technology. The AOIs of sneakers were first identified, and then the effects of sneaker design features, genders, and professional backgrounds on human visual behavior and preference were discussed. It is found that the shoelace and vamp AOIs of the sneakers were more attractive than the other AOIs. Both genders, irrespective of their professional background, were more concerned about the shoelaces than vamps. Compared to males, females were more concerned about the shoelace AOI and less interested in the vamp AOI. For the effects of professional backgrounds, the professionals displayed more interest in the shoelace AOI than the non-professionals. Beyond that, the subjective evaluation showed that the sneakers with flat and round shoelaces were more preferred by the participants, and the sneakers with canvas, cow leathery, and cotton flannel materials were more preferred than the other types of vamp materials. In future studies, a large sample of subjects with different age ranges should be employed for their visual attention and marketing behaviors. In addition, to improve the design or the advertising of sneakers, the relationship among the influencing factors (more design details involving color, logo and others, gender, professional background, price, etc.), human visual attention, and their marketing behaviors should be explored as well.

## Data Availability Statement

The raw data supporting the conclusions of this article will be made available by the authors, without undue reservation.

## Ethics Statement

The studies involving human participants were reviewed and approved by Guangdong University of Technology. The patients/participants provided their written informed consent to participate in this study. Written informed consent was obtained from the individual(s) for the publication of any potentially identifiable images or data included in this article.

## Author Contributions

All authors listed have made a substantial, direct, and intellectual contribution to the work and approved it for publication.

## Conflict of Interest

The authors declare that the research was conducted in the absence of any commercial or financial relationships that could be construed as a potential conflict of interest.

## Publisher's Note

All claims expressed in this article are solely those of the authors and do not necessarily represent those of their affiliated organizations, or those of the publisher, the editors and the reviewers. Any product that may be evaluated in this article, or claim that may be made by its manufacturer, is not guaranteed or endorsed by the publisher.

## References

[B1] Berlyne D. E. (1971). Aesthetics and Psychobiology. New York: Appleton Century-Crofts, 100.

[B2] Bradley M. M. Miccoli L. Escrig M. A. Lang P. J. (2008). The pupil as a measure of emotional arousal and autonomic activation. Psychophysiology 45, 602–607. 10.1111/j.1469-8986.2008.00654.x 18282202 PMC3612940

[B3] Brownmiller S. (1984). Femininity. New York: Ballantine Books, 86.

[B4] Caswell J. (2002). Giving credence to environmental labeling of agro-food products: Using search and experience attributes as an imperfect indicator of credibility. Ecolabels and the Greening of the Food Market, Boston, Massachusetts, USA, November. Boston, MA: Tufts School of Nutrition Science and Policy.

[B5] Chai C. Bao D. Sun L. Cao Y. (2015). The relative effects of different dimensions of traditional cultural elements on customer product satisfaction. Int. J. Ind. Ergon. 48, 77–88. 10.1016/j.ergon.2015.04.001

[B6] Corbetta M. Shulman G. L. (2002). Control of goal-directed and stimulus-driven attention in the brain. Nat. Rev. Neurosci. 3, 201. 10.1038/nrn755 11994752

[B7] Darley W. K. Smith R. E. (1995). Gender differences in information processing strategies: an empirical test of the selectivity model in advertising response. J. Advertis. 24, 41–56. 10.1080/00913367.1995.10673467

[B8] Dittmar H. Beattie J. Friese S. (1995). Gender identity and material symbols: Objects and decision considerations in impulse purchases. J. Econ. Psychol. 16, 491–511. 10.1016/0167-4870(95)00023-H8826795

[B9] Fenko A. Schifferstein H. Hekkert P. (2010). Looking hot or feeling hot: what determines the product experience of warmth?. Mater. Design 31, 1325–1331. 10.1016/j.matdes.2009.09.008

[B10] Guo F. Liu W. L. Cao Y. Liu F. T. Li M. L. (2016). Optimization design of a webpage based on Kansei engineering. Human Factors Ergon. Manuf. 26, 110–126. 10.1002/hfm.20617

[B11] Herpen E. V. Trijp H. (2011). Front-of-pack nutrition labels. Their effect on attention and choices when consumers have varying goals and time constraints. Appetite 57, 148–160. 10.1016/j.appet.2011.04.011 21554909

[B12] Ho C. H. Lu Y. N. (2014). Can pupil size be measured to assess design products?. Int. J. Ind. Ergon. 44, 436–441. 10.1016/j.ergon.2014.01.009

[B13] Ho H. F. (2014). The effects of controlling visual attention to handbags for women in online shops: evidence from eye movements. Comput. Human Behav. 30, 146–152. 10.1016/j.chb.2013.08.006

[B14] Holbrook M. B. (1986). Aims, concepts, and methods for the representation of individual differences in esthetic responses to design features. J. Consum. Res. 13, 337–347. 10.1086/209073

[B15] Hollier M. P. Rimell A. N. Hands D. S. Voelcker R. M. (1999). Multi-modal Perception. BT Technol. J. 17, 35–46.

[B16] Karjalainen T. M. (2003). Strategic brand identity and symbolic design cues. in 6th Asian Design Conference. Tsukuba Japan, 2003: Journal of the Asian Design International Conference, ed Aoki H..

[B17] Kim G. W. Yun M. H. (2018). Understanding the impression of product sounds by integrating quantitative and qualitative findings. Int. J. Ind. Ergon. 3, 98–109. 10.1016/j.ergon.2017.04.002

[B18] Kim M. Lennon S. (2008). The effects of visual and verbal information on attitudes and purchase intentions in internet shopping. Psychol. Market. 25, 146–178. 10.1002/mar.20204

[B19] Laan L. Hooge I. Ridder D. Viergever M. A. Smeets P. (2015). Do you like what you see? the role of first fixation and total fixation duration in consumer choice. Food Qual. Pref. 39, 46–55. 10.1016/j.foodqual.2014.06.015

[B20] Li B. R. Wang Y. Wang K. S. (2017). A novel method for the evaluation of fashion product design based on data mining. Adv. Manuf. 5, 370–376. 10.1007/s40436-017-0201-x

[B21] Lin Y. C. Wei C. C. (2016). A hybrid consumer-oriented model for product affective design: an aspect of visual ergonomics. Hum. Factors Ergon. Manuf. 27, 17–29. 10.1002/hfm.20403

[B22] Lindsay-Prince L. (2013). “Wanna hear my voice, look at my feet!” How Female Sneaker Aficionadas Negotiate Their Femininities and Identities Within a Male-Centric Subculture. Norwich: University of East Anglia.

[B23] Matthews D. Cryer-Coupet Q. Degirmencioglu N. (2021). I wear, therefore I am: investigating sneakerhead culture, social identity, and brand preference among men. Fashion Textiles 8, 1–21. 10.1186/s40691-020-00228-3

[B24] Milosavljevic M. Cerf M. (2008). First attention then intention. Int. J. Advert. 7, 381–398. 10.2501/S0265048708080037

[B25] Nodine C. F. Locher P. J. Krupinski E. A. (1993). The role of formal art training on perception and aesthetic judgment of art compositions. Leonardo 26, 219–227. 10.2307/1575815

[B26] Osgood C. E. Suci C. J. Tannenbaum P. H. (1957). The measurement of meaning. Audio-Visual Commun. Rev. 2, 503–504.

[B27] Overmars S. Poels K. (2015). Online product experiences: the effect of simulating stroking gestures on product understanding and the critical role of user control. Comput. Human Behav. 1, 272–284. 10.1016/j.chb.2015.04.033

[B28] Park J. Delong M. Woods E. (2012). Exploring product communication between the designer and the user through eye-tracking technology. Int. J. Fashion Des. Technol. Educ. 5, 67–78. 10.1080/17543266.2011.633566

[B29] Pieters R. Warlop L. (1999). Visual attention during brand choice: the impact of time pressure and task motivation. Int. J. Res. Market. 16, 1–16. 10.1016/S0167-8116(98)00022-6

[B30] Qu Q. X. Guo F. (2019). Can eye movements be effectively measured to assess product design? Gender differences should be considered. Int. J. Ind. Ergon. 2, 281–289. 10.1016/j.ergon.2019.06.006

[B31] Sargezeh B. A. Tavakoli N. Daliri M. R. (2019). Gender-based eye movement differences in passive indoor picture viewing: an eye-tracking study. Physiol. Behav. 6, 43–50. 10.1016/j.physbeh.2019.03.023 30922820

[B32] Statista (2021a). Projected Global Retail E-Commerce Sales in 2020, by Region. Available online at: https://www.statista.com/statistics/311357/sales-of-e-commerce-worldwide-by-region/ (accessed August 9, 2021)

[B33] Statista (2021b). E-commerce Share of Total Retail in the Asia Pacific Region in 2020, by Country or Region. Available online at: https://www.statista.com/statistics/1040590/apac-e-commerce-share-of-total-retail-by-country/#statisticContainer (accessed August 9, 2021).

[B34] Wedel M. Pieters R. (2008). Eye tracking for visual marketing. Found. Trends Market. 1, 231–320. 10.1561/1700000011

[B35] Wright A. A. Lynch J. G. Jr. (1995). Communication effects of advertising versus direct experience when both search and experience attributes are present. J. Consumer Res. 21, 708–718. 10.1086/209429

[B36] Yang G. Z. Dempere-Marco L. Hu X. P. Rowe A. (2002). Visual search: psychophysical models and practical applications. Image Vis. Comput. 20, 291–305. 10.1016/S0262-8856(02)00022-720377294

[B37] Yazdanparast A. Spears N. (2012). Need for touch and information processing strategies: an empirical examination. J. Consum. Behav. 11, 415–421. 10.1002/cb.1393

[B38] Zhang J. Wedel M. Pieters R. (2009). Sales effects of attention to feature advertisements: a Bayesian mediation analysis. J. Market. Res. 46, 669–681. 10.1509/jmkr.46.5.669

